# The Complexities of Managing Acute Coronary Syndrome in a Patient With Hereditary Hemorrhagic Telangiectasia: A Case Report and Literature Review

**DOI:** 10.7759/cureus.80758

**Published:** 2025-03-18

**Authors:** Parisa Aijaz, Rafi Aibani, Katherine Mnuskin

**Affiliations:** 1 Internal Medicine, Charleston Area Medical Center, Charleston, USA; 2 Internal Medicine, West Virginia School of Osteopathic Medicine, Charleston, USA

**Keywords:** acute coronary syndrome, anticoagulation, case report, dapt, hht

## Abstract

Hereditary hemorrhagic telangiectasia (HHT) is an uncommon autosomal dominant genetic disorder characterized by mucocutaneous and gastrointestinal telangiectasias and visceral arteriovenous malformations (AVMs). Patients with HHT have an increased risk of both bleeding and arterial and venous thrombosis. Due to the increased risk of bleeding, these patients generally cannot tolerate antiplatelet or anticoagulant therapies. This poses a particular hurdle when treating acute coronary syndrome (ACS). Our case involves a 79-year-old male patient with a past medical history of HHT, major gastrointestinal bleeding (GIB), and coronary artery disease who presented with a non-ST-segment elevation myocardial infarction (NSTEMI) and acute-on-chronic anemia. Our treatment options were limited given his intolerance to antiplatelet and anticoagulant therapies which resulted in major GIB, making him a poor candidate for percutaneous coronary intervention. We consulted cardiology and treated him with beta-blockers to decrease oxygen demand, packed red blood cell (PRBC) transfusion to increase oxygen supply, and ranolazine for symptom relief. His symptoms improved and he was discharged. Four weeks later, he suffered a cardiac arrest due to ventricular fibrillation. His family chose to pursue comfort measures, and he was transferred to an inpatient hospice. By reporting this case, we aim to highlight the unique challenges faced when managing ACS in patients with HHT. We underscore the importance of mitigating the risks of coronary artery disease in these patients. Given the limited treatment options and low tolerance to treatment modalities used for ACS, early detection of HHT and implementing effective primary prevention strategies are crucial in these patients.

## Introduction

Hereditary hemorrhagic telangiectasia (HHT), also known as Osler-Weber-Rendu syndrome, is a disorder characterized by vascular defects such as visceral and cutaneous telangiectasias and arteriovenous malformations (AVMs) in the lungs, liver, or brain. It can lead to complications such as gastrointestinal bleeding (GIB), iron deficiency anemia, hemorrhage, cerebral embolic events, venous thromboembolism, pulmonary hypertension, high output heart failure, portal hypertension, biliary disease, etc. [1].

HHT is the second most common hereditary bleeding disorder worldwide [2]. The estimated prevalence of HHT is between 1:5000 and 1:8000 individuals globally, with higher rates in certain regions due to genetic inheritance [3]. While bleeding is a significant complication in HHT, these patients are also prone to thrombotic events [4]. Although the prevalence of acute coronary syndrome (ACS) in patients with HHT is not well-documented in the literature, there are established risk factors for ACS development in these individuals.

Patients with HHT have a higher risk of developing ACS, which can occur due to plaque rupture, paradoxical emboli from pulmonary AVMs, or spontaneous coronary artery dissection [4]. Patients with both ACS and HHT present unique challenges as there are no specific ACS guidelines tailored to HHT. Our patient had a known history of HHT and coronary artery disease (CAD) and presented with non-ST-segment elevation myocardial infarction (NSTEMI). We aim to highlight the challenges faced in managing such patients and the need for further studies and ACS guidelines catered specifically to patients with HHT.

## Case presentation

Our patient was a 79-year-old man who had a past medical history of HHT, daily episodes of epistaxis, major GIB, CAD, coronary artery bypass graft (CABG) surgery, cerebrovascular disease, pulmonary emboli, heart failure with mid-range ejection fraction (an ejection fraction of 45-50%), type 2 diabetes, and hypertension. He also had chronic iron deficiency anemia secondary to his recurrent episodes of daily epistaxis, bleeding from his facial telangiectasias, and frequent melanic stools, requiring iron infusions and blood transfusions. His baseline hemoglobin was between 9 and 10 g/dL (normal: 14-16 g/dL), which would decrease to 7-8 g/dL every 3-5 weeks, requiring blood transfusions. He also received outpatient iron infusions every two weeks. He presented to the emergency department due to chest pain, shortness of breath, and a significant decline in his exercise capacity. He denied orthopnea, paroxysmal nocturnal dyspnea, and pre-syncopal episodes. His symptoms were exacerbated with exertion and improved with rest.

**Figure 1:**
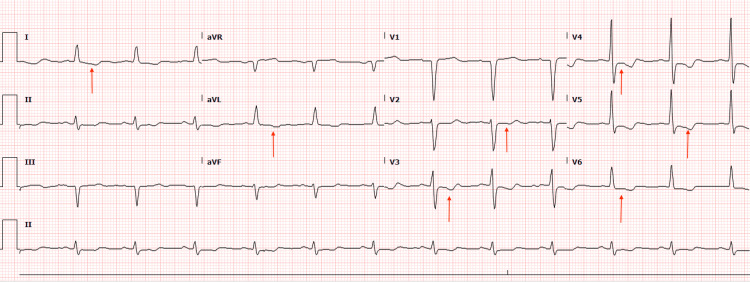
EKG of a patient with HHT showing ST depressions in the anterolateral leads 1, aVL, and V2-V6 (red arrows) EKG: electrocardiogram; HHT: hereditary hemorrhagic telangiectasia

His initial workup was significant for a hemoglobin of 7.2 g/dL, and his troponins were elevated at 141 pg/mL (reference range: ≤20 pg/mL=negative) on presentation and later peaked at 1431 pg/mL. An electrocardiogram (EKG) revealed a previously known partial left bundle branch block and new ST depressions in the anterolateral leads 1, aVL, and V2-V6 (Figure 1). He was diagnosed with NSTEMI. The patient's cardiac history was significant for CABG surgery 12 years ago. Coronary angiography (CA) three years prior reported an occluded left anterior descending (LAD) artery and right coronary artery (RCA) and 50% stenosis of the left main circumflex artery. All three grafts from the previous CABG were reportedly patent. A previous trial of antiplatelet and anticoagulation therapy had resulted in episodes of major GIB requiring multiple blood transfusions, due to which he was not currently on any antiplatelet or anticoagulation therapy. Keeping this in mind, we could not safely administer these therapies at the time of presentation.

We consulted the cardiology team; however, it was determined that any intervention would be at a very high risk of failure as the patient was not a candidate for single or dual antiplatelet therapies that are necessary after any percutaneous intervention. A shared decision was made along with the patient not to pursue invasive cardiac testing as the inherent risks of an invasive procedure outweighed the benefits for him. Additionally, he was intolerant to statins and ezetimibe due to severe myopathy. We started beta-blockers to reduce oxygen demand and administered packed red blood cells (PRBC) to improve oxygen delivery. We also started ranolazine for symptomatic relief. Echocardiography reported an ejection fraction of 45-50%. His symptoms improved with these therapies, and he was discharged home.

**Figure 2:**
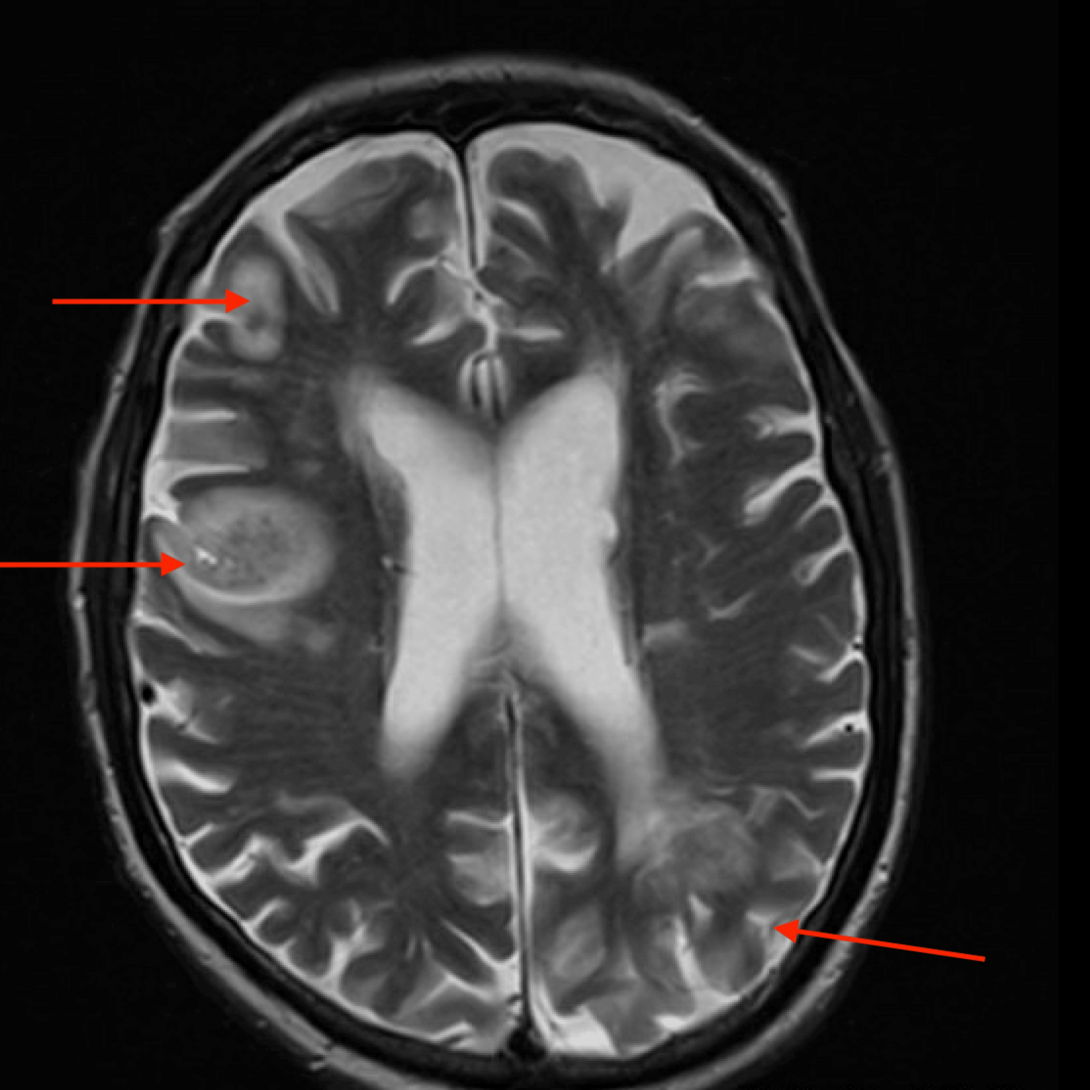
T2-weighted MRI brain showing multiple ischemic infarcts (red arrows)

Unfortunately, he suffered a cardiac arrest due to ventricular fibrillation four weeks later and was cardioverted and intubated by emergency medical services. He remained unresponsive in the intensive care unit. Brain MRI confirmed multiple bilateral infarcts, thought to have resulted from embolic showers following cardioversion (Figure 2). His family pursued comfort care, and he was transferred to an inpatient hospice. 

## Discussion

Although the prevalence of ACS in patients with HHT is not well-documented in the literature, there are established risk factors for ACS development in these individuals. ACS encompasses a range of clinical symptoms indicative of acute myocardial ischemia, including unstable angina (UA), NSTEMI, and ST-segment elevation myocardial infarction (STEMI). NSTEMI is diagnosed when the ischemia is severe enough to cause myocardial damage, leading to the release of myocardial necrosis biomarkers, such as cardiac-specific troponins T or I, or the muscle and brain fraction of creatine kinase (CK-MB), into the bloodstream [5]. The etiology can be variable, including plaque rupture, paradoxical emboli from pulmonary AVMs, or spontaneous coronary artery dissection.

HHT is a genetic condition primarily inherited in an autosomal dominant manner, marked by telangiectasias of the skin, mucous membranes, and internal organs, along with AVMs in visceral tissues [6].

Mutations in specific genes are responsible for HHT, with three genes accounting for 85% of clinical cases. HHT type 1 involves a mutation in the ENG gene coding for endoglin on chromosome 9, HHT type 2 is associated with mutations in the ACVRL1 gene coding for activin receptor-like kinase (ALK) on chromosome 12, and a combined disorder involving juvenile polyposis and HHT is linked to mutations in the MADH4 gene that codes for the transcription factor SMAD4 [7,8].

The widely accepted standard for diagnosing HHT is the Curaçao criteria, which are based on the most characteristic features of the disease. This includes spontaneous and recurrent nosebleeds, family history, telangiectasias on the skin and mucous membranes, and visceral lesions. A definitive diagnosis of HHT is made if a patient meets at least three of these four criteria. Our patient met these criteria.

About 50% of HHT patients have pulmonary AVMs, which can lead to ACS when emboli detach and migrate to the coronary arteries [9]. Another factor contributing to the increased risk of thrombosis in HHT is the propensity to develop damaged endothelial surfaces, resulting from disruptions in the regulation of the coagulation cascade [4]. These disturbances can establish a chronic prothrombotic state with subsequent consequences. Furthermore, patients with HHT often exhibit elevated levels of factor VIII and von Willebrand factor, which directly correlate with an increased risk of thrombosis [4].

For the management of ACS, current guidelines recommend the use of antithrombotic therapy involving anticoagulants and antiplatelet agents. Percutaneous coronary intervention (PCI) patients typically receive dual antiplatelet therapy (DAPT) with aspirin and a P2Y12 receptor blocker for a designated period [10]. However, patients with both ACS and HHT present a unique challenge in terms of management as there are no specific ACS guidelines tailored to HHT. Standard antithrombotic therapies, including antiplatelet agents and anticoagulants, are poorly tolerated by these patients due to an increased risk of bleeding, particularly epistaxis. The Second International Guidelines for the Diagnosis and Management of Hereditary Hemorrhagic Telangiectasia advise caution when using antiplatelet and anticoagulant agents based on patient tolerance [11].

Single antiplatelet therapy

A study by Virk et al. investigated the use of multiagent antithrombotic therapy in confirmed HHT patients to assess their risk of bleeding-related complications. The study included 119 HHT patients who were prescribed antithrombotic agents. Patients treated with single antiplatelet therapy had a slightly lower rate of dose reduction or premature medication discontinuation due to bleeding complications compared to other antithrombotic agents [2]. Additionally, the choice of antiplatelet agent was associated with different risks, with a higher percentage of reported antithrombotic treatment episodes using aspirin compared to clopidogrel in the patient cohort [2]. While these findings suggest increased tolerance with single antiplatelet agents in HHT patients, the overall analysis indicates comparable complication rates between single antiplatelet therapy and therapeutic anticoagulation. Therefore, there is insufficient evidence to support the use of single antiplatelet therapy as a replacement for the current standard of care in managing ACS or other conditions that require antithrombotic therapy in patients with HHT. Our patient had previously been on single antiplatelet therapy which resulted in a major GIB requiring multiple transfusions. Despite being off any antiplatelet or anticoagulation therapies, he had daily episodes of epistaxis, daily bleeding from his facial telangiectasia, and occasional melanotic stools and required frequent blood transfusions.

Dual antiplatelet therapy

The Second International Guidelines for the Diagnosis and Management of Hereditary Hemorrhagic Telangiectasia caution against DAPT due to the high risk of bleeding in patients with HHT. If multiple antithrombotic agents are necessary, the guidelines recommend limiting the duration of therapy and closely monitoring patients [11]. This poses challenges for the management of patients who would typically warrant DAPT after PCI or stent placement. The American College of Cardiology uses a DAPT score to assess bleeding or thrombosis risk in patients on DAPT, considering factors such as age, diabetes, prior myocardial infarction (MI) or PCI, hypertension, peripheral arterial disease, recent smoking history, heart failure, renal insufficiency, and procedural characteristics. A meta-analysis by Mihatov et al. published results in favor of the DAPT score [12]; however, this risk calculator does not account for the increased risk in patients with HHT. Clinical judgment plays a crucial role in managing such complex patients, who may require longer durations of DAPT. In highly at-risk patients, there could be some potential benefit of DAPT outweighing the bleeding risk. However, further research is needed to assess the risks of DAPT in HHT, providing better guidance for the treatment and management of these complex patients.

Anticoagulation

HHT patients generally tolerate heparin and warfarin well, but bleeding has been reported as an adverse event in some cases. Therefore, anticoagulation in HHT requires caution to balance the potential bleeding risk with the life-threatening thrombotic risks [13]. Currently, traditional anticoagulants like warfarin and heparin are the preferred choices in HHT due to their known tolerance and available reversal agents. In recent years, novel direct oral anticoagulants (DOACs) such as dabigatran, rivaroxaban, and apixaban have gained popularity for treating venous thromboembolism and non-valvular atrial fibrillation in the general population. Although bleeding is a known risk of DOACs, the incidence of severe bleeding, including intracranial bleeding, is generally lower compared to warfarin [13]. A retrospective study across HHT centers found that in 24 out of 32 treatment sessions using DOACs for atrial fibrillation or venous thromboembolism, HHT nosebleeds worsened, leading to 11 patients discontinuing the treatment. Five patients experienced severe hemorrhagic responses, necessitating hospital admissions, blood transfusions, and treatment discontinuation. Apixaban may be associated with a lower risk of bleeding problems among the new DOACs compared to rivaroxaban. Overall, the current first-choice anticoagulants in HHT are warfarin and conventional heparin due to their tolerability and available reversal agents [13]. The risks and benefits of using DOACs in HHT patients should be carefully evaluated on an individual basis, considering the potential bleeding risk and the lack of specific trial data for this patient population.

Percutaneous intervention

Patients with HHT may face challenges during CA and PCI due to factors such as underlying coronary anatomy, technical difficulties, the need for antithrombotic therapy, and an elevated bleeding risk [14-16]. A retrospective study examined the risks of CA and PCI in 665 HHT patients over 15 years. Out of the 18 patients who underwent CA, diverse indications were observed, including ACS, stable angina, heart failure, valvular disease, and pretransplant evaluation. While 13 patients had normal coronaries, aberrant findings were detected in five patients. Among HHT patients with ACS undergoing CA, major periprocedural complications occurred in three out of six patients, with two cases arising during PCI. In one patient with UA, a bare-metal stent was needed to address a specific dissection and luminal filling defect during PCI of the RCA [17].

Genetic mutations leading to endothelial damage and vessel wall fragility contribute to HHT pathophysiology, amplified by impaired transforming growth factor-beta signaling [18]. Inflammatory conditions during ACS can further increase vessel wall vulnerability, rendering HHT patients more susceptible to complications during urgent CA and PCI. Additionally, the inherent susceptibility to bleeding in HHT patients, combined with the significant bleeding risk associated with PCI and antithrombotic therapy, heightens the likelihood of complications [13]. Further research is needed to better understand the underlying pathophysiology. In conclusion, physicians must be mindful of potential procedural hazards when making decisions and preparing interventions for HHT patients presenting with ACS.

## Conclusions

ACS in patients with HHT presents a unique challenge. Mitigating the risk of CAD including optimal blood pressure, diabetes management, and weight loss is paramount in these patients. Early detection of HHT is crucial to allow the implementation of effective primary prevention strategies, given the limited options and low tolerance to treatment of ACS in these patients. Further research is needed to determine treatment strategies for ACS tailored to patients with HHT.
